# Chymase-Dependent Generation of Angiotensin II from Angiotensin-(1-12) in Human Atrial Tissue

**DOI:** 10.1371/journal.pone.0028501

**Published:** 2011-12-13

**Authors:** Sarfaraz Ahmad, Tony Simmons, Jasmina Varagic, Norihito Moniwa, Mark C. Chappell, Carlos M. Ferrario

**Affiliations:** 1 Division of Surgical Sciences, Wake Forest University School of Medicine, Winston-Salem, North Carolina, United States of America; 2 Internal Medicine/Cardiology, Wake Forest University School of Medicine, Winston-Salem, North Carolina, United States of America; 3 Hypertension and Vascular Research Center, Wake Forest University School of Medicine, Winston-Salem, North Carolina, United States of America; 4 Department of Physiology and Pharmacology, Wake Forest University School of Medicine, Winston-Salem, North Carolina, United States of America; 5 Internal Medicine/Nephrology, Wake Forest University School of Medicine, Winston-Salem, North Carolina, United States of America; Université Joseph Fourier, France

## Abstract

Since angiotensin-(1-12) [Ang-(1-12)] is a non-renin dependent alternate precursor for the generation of cardiac Ang peptides in rat tissue, we investigated the metabolism of Ang-(1-12) by plasma membranes (PM) isolated from human atrial appendage tissue from nine patients undergoing cardiac surgery for primary control of atrial fibrillation (MAZE surgical procedure). PM was incubated with highly purified ^125^I-Ang-(1-12) at 37°C for 1 h with or without renin-angiotensin system (RAS) inhibitors [lisinopril for angiotensin converting enzyme (ACE), SCH39370 for neprilysin (NEP), MLN-4760 for ACE2 and chymostatin for chymase; 50 µM each]. ^125^I-Ang peptide fractions were identified by HPLC coupled to an inline γ-detector. In the absence of all RAS inhibitor, ^125^I-Ang-(1-12) was converted into Ang I (2±2%), Ang II (69±21%), Ang-(1-7) (5±2%), and Ang-(1-4) (2±1%). In the absence of all RAS inhibitor, only 22±10% of ^125^I-Ang-(1-12) was unmetabolized, whereas, in the presence of the all RAS inhibitors, 98±7% of ^125^I-Ang-(1-12) remained intact. The relative contribution of selective inhibition of ACE and chymase enzyme showed that ^125^I-Ang-(1-12) was primarily converted into Ang II (65±18%) by chymase while its hydrolysis into Ang II by ACE was significantly lower or undetectable. The activity of individual enzyme was calculated based on the amount of Ang II formation. These results showed very high chymase-mediated Ang II formation (28±3.1 fmol×min^−1^×mg^−1^, n = 9) from ^125^I-Ang-(1-12) and very low or undetectable Ang II formation by ACE (1.1±0.2 fmol×min^−1^×mg^−1^). Paralleling these findings, these tissues showed significant content of chymase protein that by immunocytochemistry were primarily localized in atrial cardiac myocytes. In conclusion, we demonstrate for the first time in human cardiac tissue a dominant role of cardiac chymase in the formation of Ang II from Ang-(1-12).

## Introduction

It is well established that the renin-angiotensin system (RAS) is a major regulatory hormonal network influencing cardiovascular homeostasis, blood pressure, and fluid and electrolyte balance. The bioactive angiotensin peptides generated from angiotensin I (Ang I), angiotensin II (Ang II) and angiotensin-(1-7) [(Ang-(1-7)], display independent and pleiotropic biological activities *in vivo*, and interact with each other both in metabolic pathways and biological effects [1]. Biochemical and physiological studies document the existence of a tissue-based RAS within a variety of tissues including the heart [2]. More recently, a renewed interest toward the investigation of alternate mechanisms for Ang II formation upstream from Ang I arose from the work of Nagata et al. [3], who showed that Ang II could be formed by the dodecapeptide angiotensin-(1-12) [Ang-(1-12)], an extended form of Ang I. Prosser et al. [4], [5] reported that Ang-(1-12) caused vasoconstrictor effects that were abolished by inhibition of angiotensin converting enzyme (ACE) or chymase. Additional evidence for a biological action of Ang-(1-12) via Ang II formation were reported in studies in which either the peptide was administered in the rat brain [6]–[8] or following endogenous neutralization via injection of a selective Ang-(1-12) antibody [9].

Expanding on the potential functional role of this dodecapeptide as an alternate substrate for the cellular generation of angiotensin peptides, we showed that Ang-(1-12) was predominantly expressed in cardiac myocytes and proximal, distal, and collecting renal tubules of both WKY and SHR [10]. Additional studies demonstrated that Ang II formation from Ang-(1-12) did not require the presence of renin [11], [12] while others [4] showed a potential role for ACE and chymase in the cleavage of Ang-(1-12) into Ang II. A more definitive study of Ang-(1-12) in isolated cardiac myocytes from WKY and SHR found that while both ACE and chymase participated in Ang-(1-12) metabolism, a greater influence of chymase was evident in cardiac myocytes isolated from SHR [13]. This finding confirmed our previous report of increased cardiac Ang-(1-12) concentrations in SHR [10].

While the studies in rodent models and isolated cells demonstrated a functional role of endogenously expressed Ang-(1-12) in the formation of angiotensin peptides [3]–[8], [10], [11], [13]–[15], no data exists as to whether this substrate is found in humans. This study describes the existence of Ang-(1-12) in the human heart and identifies the primary enzyme accounting for Ang-(1-12) metabolism in human atrial cardio myocytes.

## Results

### Localization of Ang-(1-12) in Human Atrial Tissue

**Figure 1:**
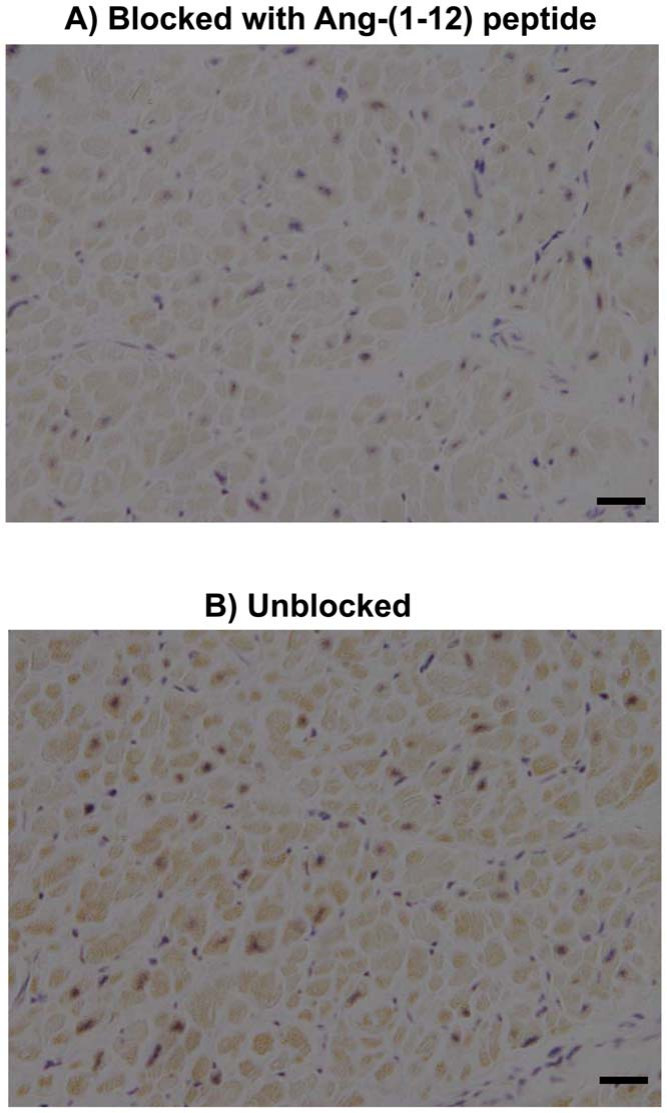
Localization of Ang-(1-12) in human atrial tissue. Comparative adjacent sections of Ang-(1-12) immunoreactivity obtained from human atrial tissue with protein A purified polyclonal antibody produced by AnaSpec. A) Antibody (1∶2,000 dilution) blocked with 100 µmol/L of human Ang-(1-12) peptide, and B) Unblocked antibody (1∶2,000 dilution). *(Magnification 400; scale bar is 50 µm)*.

Figure 1 documents the presence of immunoreactive (ir-) Ang-(1-12) products in atrial tissue collected from human subjects using a protein A purified polyclonal antibody directed to the COOH-terminus of the full length of the human Ang-(1-12) sequence. The ir-Ang-(1-12) staining is found primarily in the cytoplasm of atrial cardiomyocytes, in agreement with findings in the rat's heart [10]. Striking differences in staining of cardiomyocytes between Ang-(1-12) peptide block and unblocked tissue slides were noted (Figure 1). When viewed at higher magnification, almost all atrial cardiac myocytes showed some degree of positive staining with the Ang-(1-12) antibody (Figure 1).

### ^125^I-Ang-(1-12) Metabolism by Human Atrial Tissue

Under the conditions described in Methods, excellent separation of the radiolabeled products derived from the addition of ^125^I-Ang-(1-12) with or without the RAS inhibitor cocktail were obtained for Ang I, Ang II, Ang-(1-7), and the peaks corresponding to Ang-(1-4) fragments. ^125^I-Ang-(1-12) hydrolysis was evident in these human samples, with Ang II (69±21%; n = 9) being the predominant form found within 60 minutes after addition of ^125^I-Ang-(1-12) in the absence of all inhibitors (*No RAS inhibitors*) (Table 1). Peak areas of the chromatogram corresponding to Ang I (2±2%), Ang-(1-7) (5±2%), and Ang-(1-4) peptide fragments (2±1%) were detected in the absence of all RAS inhibitors (Table 1). ^125^I-Ang-(1-12) degradation was essentially prevented when the substrate was added to plasma membranes exposed to the cocktail of RAS inhibitors. In this condition (*All RAS inhibitors*), 98±7% of the parent ^125^I-Ang-(1-12) remained unmetabolized after 60 min incubation at 37°C (Table 1). A large amount of Ang II formation (65±18%) from the reaction mixture, whereas Ang-(1-12) remained unmetabolized (98±5%) following the removal of lisinopril only (ACE inhibitor); in this latter condition, only a small amount of Ang II formation (2±1%) was detected (Table 1).

**Table 1:** Comparative Effects of Selective Enzyme Inhibition on ^125^I-Ang-(1-12) Metabolism by plasma membrane isolated from human atrial appendage tissues.

Peptides	NO RASInhibitors	All RASInhibitors	Minus Lisinopril	Minus Chymostatin
Ang-(1-12)*	22±10^a^	98±7	98±5	31±13^a^
Ang I	2±2	1±2	ND	4±2
Ang II	69±21^a^	2±1	2±1	65±18^a^
Ang-(1-7)	5±2	ND	ND	ND
Ang-(1-4)	2±1	ND	ND	ND

HPLC of human ^125^I-Ang-(1-12) metabolic products generated by plasma membrane (50 µg) isolated from human atrial appendage incubated with or without the presence of RAS inhibitors at 37°C for 60 min. Values are expressed as % (Mean ± SEM) of Ang peptides generated from ^125^I-Ang-(1-12). *No RAS inhibitors group*: Only aminopeptidases inhibitors (amastatin & bestatin), carboxypeptidases inhibitor (benzyl succinate) and PCMB; *All RAS inhibitors group*: Above inhibitors+RAS inhibitors (lisinopril, SCH39370, MLN-4760 & chymostatin); *Minus RAS inhibitor group*: One of the RAS inhibitor (lisinopril or chymostatin) omitted at a time form the *All RAS inhibitors group*.. Results are the average of nine human samples (n = 9).

*Percent of ^125^I-Ang-(1-12) parent control remained unmetabolized after 60 min incubated with plasma membrane (50 µg) at 37°C. ND = Not detected;

a Significantly different (*P*<0.05) vs. corresponding group of *All RAS inhibitors*.

**Figure 2:**
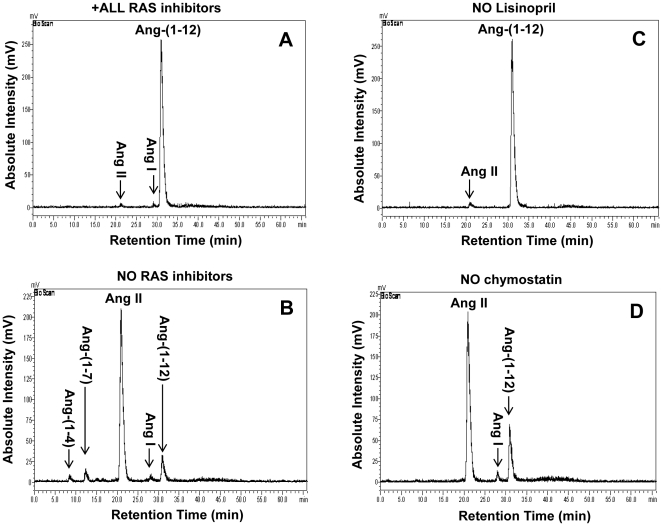
Ang-(1-12) metabolism by human atrial tissue. The metabolism of ^125^I-Ang-(1-12) by plasma membrane isolated from human atrial tissues was analyzed by HPLC coupled to an inline BioScan γ-detector. The ^125^I-Ang-(1-12) was incubated with human plasma membrane for 60 min at 37°C with or without the inhibitor cocktail and the metabolites were separated by HPLC. *A*: Chromatograms represent the hydrolysis of ^125^I-Ang-(1-12) in the presence of all RAS inhibitors (*All RAS inhibitor group* containing lisinopril, SCH39370, MLN-4760, chymostatin, bestatin, amastatin, benzyl succinate, and PCMB). *B*: Hydrolysis of ^125^I-Ang-(1-12) in the absence of all RAS inhibitors cocktail (*No RAS inhibitors group* containing only bestatin, amastatin, benzyl succinate, and PCMB). *C*: Hydrolysis of ^125^I-Ang-(1-12) in the presence of the inhibitor cocktail that lacks only Lisinopril (*No lisinopril inhibitor group* containing all inhibitors as described in “*A*” except ACE inhibitor). *D*: Hydrolysis of ^125^I-Ang-(1-12) in the presence of inhibitor cocktail that lacks only chymostatin (*No chymostatin group* containing all inhibitors as described in “*A*” except chymase inhibitor). Before adding the ^125^I-Ang-(1-12), the plasma membrane was pre-incubated with inhibitors (each added at a dose of 50 µM) for 15 min at 37°C. The arrow indicates the retention time of ^125^I-Ang-(1-12) and its metabolic products. HPLC results are representative of three or more separate metabolism experiments for each human sample.

The chromatograms depicting the effect of peptidase inhibition on ^125^I-Ang-(1-12) metabolism are shown in Figure 2-panels A to D. In the presence of all RAS inhibitors, the chromatogram shows a large peak area of unmetabolized Ang-(1-12) along with very small peaks of Ang I and Ang II (Figure 2-panel A), whereas in the absence of all RAS inhibitors the peak area of unmetabolized Ang-(1-12) is a small fraction of that observed in the presence of all inhibitors. The major peak corresponds to Ang II while peaks corresponding to the synthetic forms Ang I, Ang-(1-7), and Ang-(1-4) are expressed minimally in the absence of all RAS inhibitors (Figure 2-panel B). Ang peptides formation from ^125^I-Ang-(1-12) is only 2±1% (Ang II) when only lisinopril is removed from the cocktail of inhibitors (Figure 2-panel C), whereas the selective removal of chymostatin from the cocktail of inhibitors has a diametrically opposite effect. As shown in Figure 2-panel D, Ang II formation from ^125^I-Ang-(1-12) metabolism represents now the major peak accounting for 65±18%. Removal of chymostatin is also associated with the presence of a minor peak for Ang I (4±2%). The proportion of Ang II produced from ^125^I-Ang-(1-12) in the absence of chymostatin (Ang II formation 65±18%; *P*<0.05; n = 9) was not significantly different than that observed when ^125^I-Ang-(1-12) was incubated with human atrial membranes in the absence of all inhibitors (Ang II formation 69±21%; n = 9) (Table 1).

### Relative Contributions of ACE or Chymase in Human ^125^I-Ang-(1-12) Metabolism

**Figure 3:**
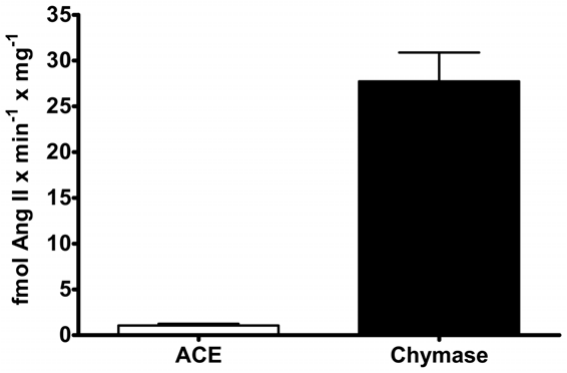
Chymase and ACE contribution to generate Ang II from Ang-(1-12). Chymase and ACE enzyme mediated generation of Ang II product from the metabolism of ^125^I-Ang-(1-12) (1 nmol/L) by plasma membrane isolated from human atrial tissues. The contribution of each enzyme (chymase, and ACE) activity (fmol Ang II formation×min^−1^×mg^−1^) were calculated based on Ang II product analysis by HPLC in each human samples incubated with or without the chymostatin and lisinopril (each added at a dose 50 µM) for 60 min at 37°C. Data are expressed as mean ± SEM; total n = 9 (4 female).

Based on the Ang II product generated from ^125^I-Ang-(1-12) under different combinations of RAS inhibitors, Figure 3 shows the relative expression of Ang II-forming by ACE and chymase activities in the plasma membranes isolated from human atrial tissue for both male and female samples. All samples, regardless of sex, expressed significantly high chymase contribution for Ang II generation (28±3.1 fmol Ang II formation×min^−1^×mg^−1^) as compared to a much lower or non-detectable contribution of ACE in forming Ang II (1.1±0.2 fmol Ang II formation×min^−1^×mg^−1^) from Ang-(1-12).

To reassure ourselves that the results obtained with the chymostatin inhibitor reflected a selective inhibition of chymase, we further evaluated the hydrolysis of the colorimetric substrate (*N*-Succinyl-Ala-Ala-Pro-Phe-*p*-nitroanilide; *N*-suc-AAPF-pNA) by human plasma membrane in the absence and in the presence of chymostatin. The pattern of *N*-suc-AAPF-pNA hydrolysis was consistent with the chymase activity detected by the HPLC for Ang II formation from ^125^I-Ang-(1-12). Again the hydrolysis of *N*-suc-AAPF-pNA was completely blocked in the presence of chymostatin in the reaction mixtures. For final verification of our conclusions, ^125^I-Ang-(1-12) metabolism was evaluated in the presence of the chymase specific inhibitor (TEI-F00806) [18]. In this study, the hydrolysis of Ang-(1-12) by human plasma membrane was prevented by 95% in the presence of TEI-F00806 (100 µM).

### Chymase Expression in Human Atrial Tissue

**Figure 4:**
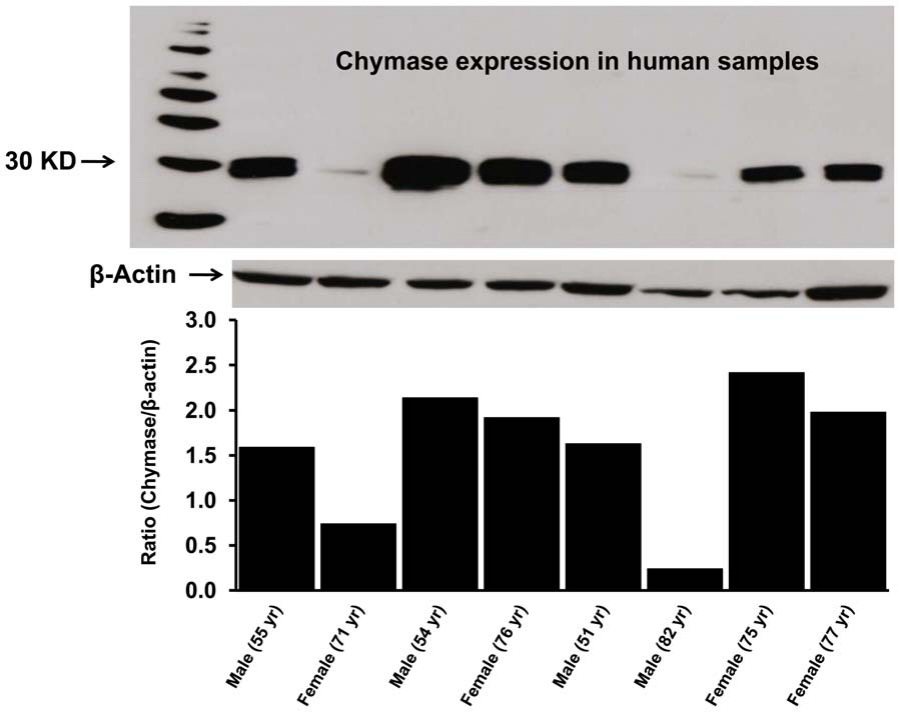
Chymase protein expression in human atrial tissue. Chymase protein expression in human atrial samples was analyzed by western blotting using a primary monoclonal anti-human chymase antibody (CMA1 antibody from R&D System, Cat# MAB-4099; 2 µg/mL). The plasma membrane (50 µg protein) were separated by gel electrophoresis and transferred on PVDF. Equal protein loading on each lane was confirmed by β-actin detection (1∶5,000 dilution). Level of protein expression was shown as relative O.D. ratio (chymase/β-actin) for each human sample.

Chymase protein expression, determined in 8 of the 9 human samples using a primary monoclonal anti-human chymase antibody, showed an immunoreactive band (∼30 KD) for chymase protein (Figure 4). The ir-chymase bands were normalized with β-actin and relative chymase protein expression in the human atrial tissue was consistence with the HPLC results of chymase activity to generate Ang II products from ^125^I-Ang-(1-12) (Figure 3). In addition, a significant correlation was found between chymase activity and protein expression (r = 0.954, P<0.002).

**Figure 5:**
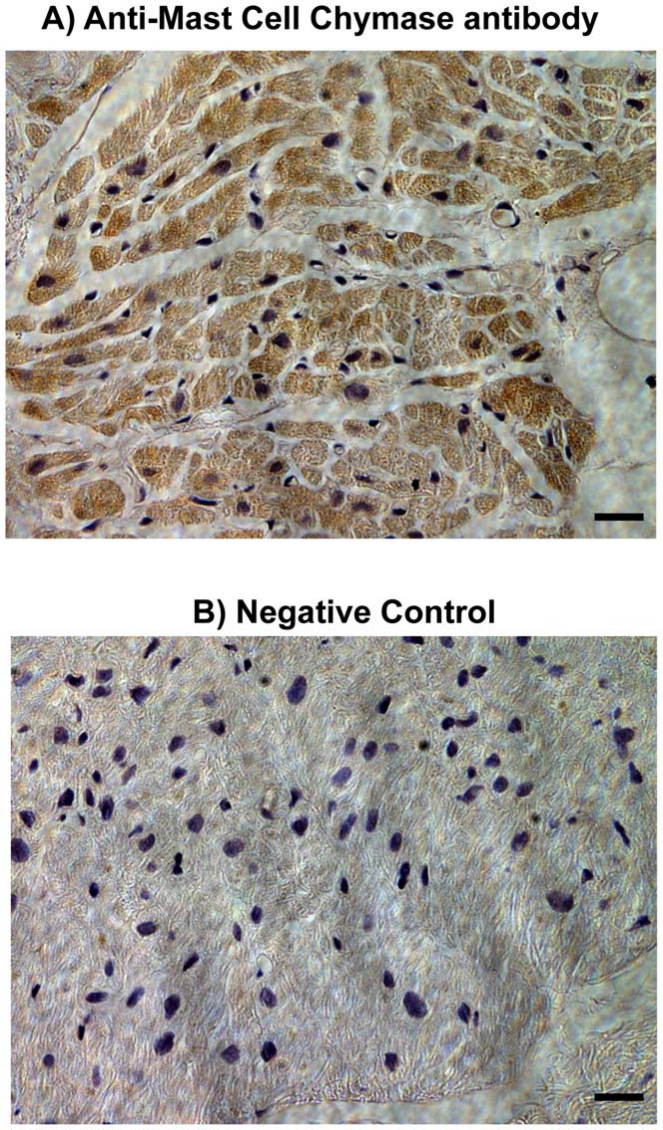
Immunohistochemistry of human atrial tissue for chymase. Immunostaining of human atrial tissue using an Anti-Mast Cell chymase antibody (Abcam Inc., Cambridge, MA; Cat# ab2377) revealed high expression of chymase within atrial cardiac myocytes (A). Negative control without primary antibody shows no staining for chymase (B). *(Magnification 400; scale bar is 50 µm)*.

Immunolocalization of chymase was also analyzed in these atrial tissues by using an anti-mast cell chymase monoclonal antibody. As illustrated in Figure 5, a strong immunostaing for the presence of chymase was detected within the cardiac myocytes of atrial tissue. Negative controls without primary antibody show no staining for chymase (Figure 5).

## Discussion

We show that human atrial tissue samples obtained from patients undergoing cardiac interventions for correction of atrial fibrillation expresses ir-Ang-(1-12) and converts ^125^I-Ang-(1-12) into angiotensin peptides. The study also demonstrates that chymase is the main metabolic pathway by which this extended form of Ang I serves as a substrate for the generation of Ang II in human atrial samples. Detection of ir-Ang-(1-12) in human atrial tissue and the capability of this tissue to convert this extended form of Ang I into Ang II are in keeping with other studies in which Ang II was the predominant angiotensin peptide formed from ^125^I-Ang-(1-12) in the isolated perfused heart of three normotensive and two hypertensive rat strains [12] and in neonatal cardiac myocytes from WKY and SHR [13]. The very low levels of Ang-(1-7) found at the time when either all inhibitors were present or only the chymase inhibitor was removed suggest a lower abundance in these tissues of either ACE2 or other peptidases known to contribute to the generation of Ang-(1-7) from Ang II [1], [17], [18]. It is also possible that the presence of the aminopeptidase inhibitors, amastatin and bestatin, prevented further conversion of Ang II into smaller fragments.

^125^I-Ang-(1-12) was cleaved efficiently in the absence of the chymostatin inhibitor to values not different than those found when Ang-(1-12) was incubated in the absence of any of the inhibitors. In both conditions, labeled Ang II was the predominant product detected 60 minutes after addition of ^125^I-Ang-(1-12). The human Ang-(1-12) sequence is H-Asp^1^-Arg^2^-Val^3^-Tyr^4^-Ile^5^-His^6^-Pro^7^-Phe^8^-His^9^-Leu^10^-Val^11^-Ile^12^-OH. Since the chymotryptic cleavage sites for human chymase are the Tyr^4^-Ile^5^ and Phe^8^-His^9^, these data suggest that chymase generates Ang II by direct hydrolysis of the Phe^8^-His^9^ bond of Ang-(1-12). This interpretation agrees with the finding that in our experiments Ang I formation represented less than 2% of the Ang-(1-12) hydrolysis.

A critical role for chymase in accounting for the processing of Ang-(1-12) is reinforced through the corresponding consistency of the chymase activity measured in each of the human samples. Since the atrial tissue employed in these studies was obtained from subjects undergoing cardiac surgery for the treatment of heart rhythm disorders (Table 2), we cannot exclude the possibility that the relatively high chymase activity reflects increased expression of this enzyme in this condition. Although an increase in cardiac chymase activity has been reported in both clinical and experimental forms of heart failure [19]–[23], several studies suggest that in humans Ang II formation from Ang I is primarily dependent upon chymase rather than ACE [19], [20], [22], [24]–[27]. A comparative study by Akasu et al. [28] in human, hamster, rat, rabbit, dog, pig, and marmoset tissues demonstrated a primary role of lung ACE in all species but human cardiac and lung tissues. In these tissues, a chymase-like activity solely accounted for the Ang II-forming activity. Balcells et al. [29] reported that chymase rather than ACE was the predominant enzyme accounting for intracardiac Ang II generation in humans, whereas Ang II forming activity from ACE was higher in cardiac tissue obtained from mouse, dog, rabbits, rat, and mice. The rodent heart might be an exemption since Wei et al. [27] showed that mast-cell derived chymase contributes to Ang II from Ang I. In their studies the content of Ang II in left ventricular interstitial fluid was not altered by high doses of an ACE inhibitor but was suppressed in the presence of a chymase inhibitor [27]. It is possible that upregulation of chymase expression or activity may occur in conditions of ACE inhibition, since chronic ACE inhibition causes a marked bradykinin/B2 receptor-mediated increase in cardiac interstitial fluid chymase activity that does not occur in mast cell-deficient KitW/KitW-v mice [27]. Becari et al. [30] reported an 80% inhibition of Ang I induced carotid artery vasoconstriction by the administration of chymostatin in enalapril-treated SHR rats. Since the atrial tissues were obtained from 7 of the 9 subjects not treated with ACE inhibitors and none were treated with Ang II receptor blockers (Table 2), our findings imply a major and independent role of cardiac chymase in the biotransformation of ^125^I-Ang-(1-12) to angiotensin peptides.

**Table 2:** Diagnosis, drug treatment and clinical statement of human heart patients.

PatientSex/age)	Diagnosis	SurgicalProcedure	ACEInhibitors	ARB	β-Blocker
Male/55 yr	MV defect	MAZE+MV repair	No	No	No
Male/54 yr	AF	MINI-MAZE	No	No	Yes
Male/51 yr	AF	MINI-MAZE	No	No	Yes
Male/54 yr	ASD+AF	MAZE+ASD repair	No	No	No
Male/82 yr	CAD	MAZE+CBS	No	No	Yes
Female/71 yr	AF	MINI-MAZE	Yes	No	Yes
Female/76 yr	AF	MINI-MAZE	No	No	Yes
Female/75 yr	AF	MAZE	Yes	No	Yes
Female/77 yr	AF	MINI-MAZE	No	No	Yes

Abbreviation: MV, mitral valve; AF, atrial fibrillation; CAD, Coronary artery disease; ASD, Atrial septal defect; CBS, Cardiac bypass surgery; ACE, angiotensin converting enzyme, and ARB, angiotensin receptor blocker.

Our results and those discussed above indicate that species differences may influence the contribution of ACE and chymase to cardiac Ang II formation. Urata et al. [24]–[26], [31] first showed in human cardiac ventricles that formation of Ang II by a serine proteinase-dependent enzyme accounted for 80% of Ang II formation while less than 10% of the Ang II produced depended upon ACE. Later studies showed that this serine proteinase was structurally characterized as a human member of the chymase group of enzyme which is not inhibited by ACE inhibitors [24]–[26], [31], [32]. According to Fleming [33], chymase functions as a tissue Ang II-forming enzyme in human heart, arteries, and lungs while ACE accounts for the majority of Ang II formation from Ang I in the circulation. Chymase broad functional activities includes its participation in collagen IV turnover [34], hydrolysis of apolipoprotein E [35], thrombin, and fibrin [36], [37].

Although numerous studies confirmed that chymostatin is a specific chymase inhibitor [4], [5], [13], [16], [20], [28], [29], [32], [38]–[45], this inhibitor may also nonspecifically reduces the activity of cathepsin G, cysteine proteases, and high-molecular weight proteasomes [46]. A recent report suggested that elastase-2, primarily expressed in human and mouse epidermis may represent an alternate pathway for Ang II generation from Ang I in conditions of chronic ACE inhibition in rats but not human vascular tissue [30]. While our past studies did not exclude a potential contribution of a chymostatin-sensitive elastase-2 to Ang-(1-12) metabolism in the rat [13], our current findings in human atrial myocytes suggest that human elastase does not account for our findings. This interpretation is based in part on the demonstration that chymostatin inhibited the hydrolysis of the colorimetric *N*-suc-AAPF-pNA substrate by human plasma membranes. Although *N*-suc-AAPF-pNA is not hydrolyzed by human leukocyte elastase [47], this finding did not exclude a contribution of human pancreatic elastase. Moreover, our additional finding that the orally active chymase inhibitor (TEI-F00806) [16] prevented the hydrolysis of Ang-(1-12) by 95% strengthens the importance of cardiac chymase as the primary enzyme accounting for Ang-(1-12) metabolism in human samples. This interpretation is further reinforced by the concurrent measurements of chymase protein, a strong correlation between chymase activity and expression, and the detection of chymase immunoreactive products in the atrial human tissue.

While studies of Ang-(1-12) processing in cardiac myocytes from neonatal rats showed a contribution of ACE and neprilysin to Ang-(1-12) metabolism in WKY, an increase in cardiac chymase activity in Ang-(1-12) metabolism was found in SHR [13]. Prosser et al. [4], [5] reported that both ACE and chymase contributed to Ang-(1-12) metabolism in the vascular system and the heart of Sprague Dawley rats. Since the Ang II generating capacity from Ang-(1-12) by ACE appeared to be predominantly found in the vasculature, these data suggest tissue specific differential roles for ACE and chymase in Ang-(1-12) metabolism.

In summary, the demonstration of ir-Ang-(1-12) in human atrial samples and the characterization of its degradation by chymase now paves the way for a further exploration of the role of this alternate substrate in the regulation of cardiac function and disease. Identification of Ang-(1-12) in human samples is a necessary prerequisite for further research since in the past an extensive characterization of the synthetic tetradecapeptide Ang-(1-14) as a source for Ang II formation met with failure because it was found not to be expressed in human subjects [48], [49]. On the other hand, a recent study by Bujack-Gizycka [14] using mass spectrometry coupled with liquid chromatography showed ex-vivo formation of Ang-(1-12) from synthetic tetradecapeptide Ang-(1-14) in the rat aorta. In their study, Ang-(1-14) was rapidly converted to Ang-(1-12) and subsequent formation of Ang II, Ang-(1-7), and Ang-(1-9) [14]. These data are in keeping with our previous demonstration of non-renin dependent pathways for Ang-(1-12) metabolism [11], [12]. As discussed by Dell'Italia et al. [21], [22], [50], non-ACE formation of Ang II is known to maintain plasma and cardiac Ang II concentrations in heart failure subjects treated with adequate doses of ACE inhibitors. The present studies revealed a tissue pathway that may account for Ang II formation in the heart through the direct conversion of Ang-(1-12) into Ang II.

## Materials and Methods

### Ethics Statement

The study has been approved by the Wake Forest University Medical Center (IRB# 00004355; approved on November 30th 2010). The human samples were collected from the patients undergoing cardiac surgery (Table 2) at the Wake Forest University Baptist Medical Center (Winston-Salem, NC, USA). The Wake Forest Medical Center committee overseeing ethical standards and regulatory compliance in human research at Wake Forest School of Medicine specifically determined that consent was not necessary for this study.

### Reagents

Human angiotensin-(1-12) (>99% purity) was purchased from GenScript USA Inc. (Piscataway, NJ). Lisinopril (ACE inhibitor) and SCH39370 (neprilysin inhibitor) were obtained from Merck Inc. (West Point, PA). MLN-4760 (ACE2 inhibitor) was obtained from Millennium Pharmaceuticals (Cambridge, MA). Chymostatin (chymase inhibitor), amastatin, bestatin, benzyl succinate and *p*-chloromercuribenzoate (PCMB), *N*-suc-AAPF-pNA were purchased from Sigma-Aldrich Co. (St. Louis, MO). The TEI-F00806 (a chymase specific inhibitor) was a gift from Teijin Pharma (Tokyo, Japan). ^125^Iodine was purchased from PerkinElmer Life and Analytical Sciences, Inc. (Waltham, MA). All other chemicals used in this study were of analytical grade and were obtained from Sigma (St. Louis, MO) and Fisher Scientific (Atlanta, GA).

### Human Tissue (atrial appendage)

Human left atrial appendage tissue from 5 men (Mean ± SD, 59±13 yrs.) and 4 women (Mean ± SD, 75±3 yrs.) were obtained from patients undergoing cardiac surgery at Wake Forest University Baptist Medical Center (Winston-Salem, NC). All patients underwent surgical excision of the left atrial appendage (LAA) as a routine part of the MAZE surgical procedure [51]. Diagnosis, drug treatment and clinical status of all nine patients are described in Table 2. The excised LAA tissue obtained from these patients was kept in cold cardioplegic solution from the time of removal, frozen within 15–30 minutes, and stored at −80°C until metabolism studies. The use of these tissues was approved by the Wake Forest University Medical Center Institutional Review Board.

### Histology and Immunohistochemistry

Left atrial tissues from human cardiac patient were fixed in 4% paraformaldehyde for 24 h and then transferred into 70% ethanol. After dehydration, the tissues were embedded in paraffin and each section was cut in 5-µm thick sections. Immunohistochemistry was performed using well characterized protein A purified polyclonal antibody directed to the COOH-terminus of full length of human Ang-(1-12) sequence [Asp^1^-Arg^2^-Val^3^-Tyr^4^-Ile^5^-His^6^-Pro^7^-Phe^8^-His^9^-Leu^10^-Val^11^-Ile^12^] (AnaSpec, San Jose, CA). The binding affinity and cross-reactivity of protein A purified antibody (IgG) was checked for human Ang-(1-12) and small Ang peptides [Ang I, Ang II and Ang-(1-7)]. The binding affinity of this antibody for human Ang-(1-12) was very high (EC_50_ = 34.7±2.0 fM) as compared to Ang I (EC_50_ = 26.2±8.6 pM), Ang II (EC_50_ = 0.28±0.02 nM) and Ang-(1-7) (EC_50_ below detection). The antibody was independently used to detect immunoreactive Ang-(1-12) in human atrial tissue using the avidin-biotin horseradish peroxidase techniques as previously reported by our laboratory [10], [11]. Briefly, slides were deparaffinized in xylene (15 min), subsequently dipped in ethanol (100%, 95% and 75%, respectively) and washed in double distilled water. The clear slides were incubated in 3% hydrogen peroxide to block the endogenous peroxidase. The sections were first blocked by 5% normal goat serum in phosphate buffered saline (PBS) for 30 min at room temperature and then incubated with protein A purified human Ang-(1-12) primary antibody (1∶2000 dilution in 1% BSA in PBS with 2% normal goat serum) for overnight at 4°C. Sections, independently treated with 5% normal goat serum in the absence of the primary antibody, served as negative controls. Additional control included sections treated with the primary antibody preincubated with 100 µmol/L of the human Ang-(1-12) peptide to which the antibodies were directed.

After incubating in primary antibody, each section was washed three times in PBS and incubated with biotinylated goat anti-rabbit secondary antibody (1∶400 dilution in 1% BSA in PBS with 2% normal goat serum; Vector Laboratories Inc., Burlington, CA) for 3 hrs. After washing the secondary antibody with PBS, sections were stained with 3,3′-diaminobenzidine (DAB, Sigma-Aldrich Chemical Co. St. Louis, MO) in Tris-buffered saline (0.05 mol/L, pH 7.6–7.7), and counterstained with hematoxylin before being dehydrated and mounted. Photographs of the resultant immunoreactive staining were acquired using a bright-field Nikon microscope system (Melville, NY), including a Diagnostic Instruments Digital SPOT RT, three-pass capture, thermoelectrically cooled charged-coupled camera (Sterling Heights, MI), and processed using the SPOT Advanced software.

Immunohistochemistry of chymase expression in human atrial tissue slides was also performed as describe above. The atrial tissue slides were incubated for 20 hrs at 4°C with Anti-mast cell chymase mouse monoclonal primary antibody (1∶100 dilution; Abcam, Cambridge, MA; Catalog# ab2377). The PBS washed slides were then treated with biotinylated goat anti-mouse secondary antibody (1∶200 dilution; Vector Laboratories Inc., Burlington, CA) for 60 min at room temperature. For negative control, in place of primary antibody, slides were treated with only normal goat serum.

### Plasma membrane preparation

Membranes were prepared at 4°C in a manner similar to the approaches previously described by our laboratory [52] and by Urata et al. [26] Frozen atrial tissue (50–100 mg) was homogenized at 4°C in 1 mL PBS (pH 7.4) in a Qiagen TissueLyzer (Valencia, CA). The homogenate was centrifuged at low spin (200 g) for 5 min at 4°C to remove the connective tissues and cell debris. The supernatant was transferred into a new tube and centrifuged at 28,000 g for 20 min at 4°C to collect the plasma membrane. The pellet was resuspended in PBS and centrifuged as described above. Finally, the pellet was resuspended in PBS and stored at −80°C till its use for metabolism studies. The concentration of each cardiac membrane preparation is expressed in terms of protein concentration measured by Bradford Reagent using bovine serum albumin (BSA) as the standard; the results were normalized in terms of per mg protein.

### ^125^I-Ang-(1-12) Metabolism by Human Plasma Membranes

Metabolism of human Ang-(1-12) by plasma membrane isolated form atrial tissue was studied under different combinations of RAS inhibitors as documented in Table 3.

**Table 3:** Outline of enzyme inhibitors employed in the experiments.

Group	Inhibitors added (50 µM each)
*No RAS inhibitor group*	Only peptidases inhibitors (amastatin, bestatin & benzyl succinate) and P-chloromercuribenzoate (PCMB).
*All RAS inhibitor group*	Above inhibitors+RAS inhibitors [lisinopril for ACE, MLN-4760 for ACE2, SCH39370 for NEP and chymostatin for chymase].
*Minus RAS inhibitor groups*	All above inhibitors except one of the RAS inhibitor (lisinopril or chymostatin) was omitted at a time from the reaction mixture.

For ^125^I-Ang-(1-12) metabolism studies, plasma membranes (50 µg per reaction mixture) were preincubated for 15 min under various combinations of RAS and peptidases inhibitors (50 µM each). After preincubation of plasma membranes with the inhibitors, human ^125^I-Ang-(1-12) (1 nmol/L; specific activity 3,900 cpm/fmol) was added to the reaction medium and incubated for an additional 60 min at 37°C. At the end of the incubation time, the reaction was stopped by adding equal volume of 1% phosphoric acid, mixed well, and centrifuged at 28,000 g for 20 min to remove the plasma membrane. The clear supernatants were stored at −20°C until processing the samples for Ang contents [Ang-(1-12), Ang I, Ang II and Ang-(1-7)] by HPLC analysis. On the day of HPLC analysis, the samples were filtered before separation by reverse-phase high-performance liquid chromatography. We used a linear gradient from 10% to 50% mobile phase B at a flow rate of 0.35 mL/min at ambient temperature. The solvent system consisted of 0.1% phosphoric acid (mobile phase A) and 80% acetonitriles/0.1% phosphoric acid (mobile phase B). The eluted ^125^I products were monitored by an in-line flow-through gamma detector (BioScan Inc., Washington, DC). Products were identified by comparison of retention time of synthetic [^125^I] standard peptides and the data were analyzed with Shimadzu LCSolution (Kyoto, Japan) acquisition software. The iodination of human Ang-(1-12) and other angiotensins were performed as described previously [53].

### Contribution of Specific Enzyme (ACE or Chymase)

The contribution of specific enzyme (ACE or chymase) to ^125^I-Ang-(1-12) hydrolysis were analyzed by measuring the amount of Ang products generated after exposing ^125^I-Ang-(1-12) with plasma membrane in the presence of all RAS inhibitors cocktail and in the absence of specific enzyme inhibitors for ACE or chymase only (as described above). Supernatants were collected after exposing the ^125^I-Ang-(1-12) in the presence of the plasma membrane for 60 min at 37°C under two different enzyme inhibitors conditions (+All RAS inhibitors *versus* minus ACE/chymase inhibitor only) and were analyzed by HPLC as described above. The enzyme activity was calculated based on adding 1 nmol/L of ^125^I-Ang-(1-12) substrate to the reaction mixture and determining the amount of Ang II product formation. Experiments were performed three or more times and the enzyme activity values were reported as fmoles of Ang II product formation from ^125^I-Ang-(1-12) substrate per min per mg protein (fmol Ang II formation×min^−1^×mg^−1^).

Chymase activity was also monitored by evaluating the hydrolysis of 1 mM *N*-suc-AAPF-pNA in the plasma membrane of six human atrial tissues. Briefly, all assays were performed in a total reaction volume of 200 µL in the 96-well microtiter plates containing plasma membranes with or without the presence of chymostatin (chymase inhibitor; 50 µM) as described above in 50 mM Tris buffer (pH 8.0) containing 1.5 M NaCl and 1% dimethylsulphoxide. The increase in absorbance at 410 nm was measured at 37°C for different time points (0–180 min) for the released p-nitroaniline. In addition, Ang II formation from ^125^I-Ang-(1-12) was also investigated in the absence and in the presence of TEI-F00806 (100 µM; an orally active chymase-specific inhibitor provided by Teijin Pharma, Tokyo, Japan). Briefly, plasma membranes isolated from human atrial tissue (50 µg per reaction mixture) and ^125^I-Ang-(1-12) were incubated in the presence or absence of TEI-F00806 (as described above for chymostatin) for 37°C for 60 min. At the end of incubation, samples were analyzed by HPLC for Ang II formation from ^125^I-Ang-(1-12).

### Western blot Analysis of Human Chymase

The expression of chymase protein in human plasma membrane samples were assessed by immunoblot technique as previously described [54]. Briefly, the plasma membranes (50 µg protein) were separated by gel electrophoresis (10% gel) and transferred to polyvinylidene defluoridated membranes (PVDF). The PVDF membranes were probed with a primary monoclonal anti-human chymase antibody (CMA1 antibody from R&D System, Minneapolis, MN, Cat# MAB4099; 2 µg/mL) and mouse anti-β-actin (1∶5,000; Sigma–Aldrich, St. Louis, MO, Cat# A-5441). After incubation with the primary antibody, the membranes were probed with the horseradish peroxidase-conjugated secondary antibody (anti-mouse, 1∶5,000; Pierce Inc., Rockford, IL, USA). Immune complexes were visualized using ECL plus detection reagents (Pierce). Densitometric analysis was performed by measuring the intensity of all Western Blotting bands with the MCID imaging system (MCID Elite 7.0, Imaging Research Inc., St. Catharines, ON, Canada).

### Statistical Analysis

Experiments were repeated independently three or more times. All values are reported as means ± SEM. The Student's *t*-test and repeated-measures ANOVA followed by a Turkey's post hoc test for multiple comparisons were used to determine significant differences at *P*<0.05 using GraphPad Prism 5.0 software (San Diego, CA).

## References

[1] Ferrario CM (2010). New physiological concepts of the renin-angiotensin system from the investigation of precursors and products of angiotensin I metabolism.. Hypertension.

[2] Paul M, Poyan MA, Kreutz R (2006). Physiology of local renin-angiotensin systems.. Physiol Rev.

[3] Nagata S, Kato J, Sasaki K, Minamino N, Eto T (2006). Isolation and identification of proangiotensin-12, a possible component of the renin-angiotensin system.. Biochem Biophys Res Commun.

[4] Prosser HC, Forster ME, Richards AM, Pemberton CJ (2009). Cardiac chymase converts rat proAngiotensin-12 (PA12) to angiotensin II: effects of PA12 upon cardiac haemodynamics.. Cardiovasc Res.

[5] Prosser HC, Richards AM, Forster ME, Pemberton CJ (2010). Regional vascular response to ProAngiotensin-12 (PA12) through the rat arterial system.. Peptides.

[6] Arakawa H, Chitravanshi VC, Sapru HN (2011). The hypothalamic arcuate nucleus: a new site of cardiovascular action of angiotensin-(1-12) and angiotensin II.. Am J Physiol Heart Circ Physiol.

[7] Arnold AC, Isa K, Shaltout HA, Nautiyal M, Ferrario CM (2010). Angiotensin-(1-12) requires angiotensin converting enzyme and AT1 receptors for cardiovascular actions within the solitary tract nucleus.. Am J Physiol Heart Circ Physiol.

[8] Chitravanshi VC, Sapru HN (2011). Cardiovascular responses elicited by a new endogenous angiotensin in the nucleus tractus solitarius of the rat.. Am J Physiol Heart Circ Physiol.

[9] Isa K, Garcia-Espinosa MA, Arnold AC, Pirro NT, Tommasi EN (2009). Chronic immunoneutralization of brain angiotensin-(1-12) lowers blood pressure in transgenic (mRen2)27 hypertensive rats.. Am J Physiol Regul Integr Comp Physiol.

[10] Jessup JA, Trask AJ, Chappell MC, Nagata S, Kato J (2008). Localization of the novel angiotensin peptide, angiotensin-(1-12), in heart and kidney of hypertensive and normotensive rats.. Am J Physiol Heart Circ Physiol.

[11] Ferrario CM, Varagic J, Habibi J, Nagata S, Kato J (2009). Differential regulation of angiotensin-(1-12) in plasma and cardiac tissue in response to bilateral nephrectomy.. Am J Physiol Heart Circ Physiol.

[12] Trask AJ, Jessup JA, Chappell MC, Ferrario CM (2008). Angiotensin-(1-12) is an alternate substrate for angiotensin peptide production in the heart.. Am J Physiol Heart Circ Physiol.

[13] Ahmad S, Varagic J, Westwood BM, Chappell MC, Ferrario CM (2011). Uptake and metabolism of the novel peptide angiotensin-(1-12) by neonatal cardiac myocytes.. PLoS One.

[14] Bujak-Gizycka B, Olszanecki R, Suski M, Madek J, Stachowicz A (2010). Angiotensinogen metabolism in rat aorta: robust formation of proangiotensin-12.. J Physiol Pharmacol.

[15] Varagic J, Trask AJ, Jessup JA, Chappell MC, Ferrario CM (2008). New angiotensins.. J Mol Med (Berl).

[16] Maeda Y, Inoguchi T, Takei R, Sawada F, Sasaki S (2010). Inhibition of chymase protects against diabetes-induced oxidative stress and renal dysfunction in hamsters.. Am J Physiol Renal Physiol.

[17] Ferrario CM, Varagic J (2010). The ANG-(1-7)/ACE2/mas axis in the regulation of nephron function.. Am J Physiol Renal Physiol.

[18] Welches WR, Brosnihan KB, Ferrario CM (1993). A comparison of the properties and enzymatic activities of three angiotensin processing enzymes: angiotensin converting enzyme, prolyl endopeptidase and neutral endopeptidase 24.11.. Life Sci.

[19] Batlle M, Roig E, Perez-Villa F, Lario S, Cejudo-Martin P (2006). Increased expression of the renin-angiotensin system and mast cell density but not of angiotensin-converting enzyme II in late stages of human heart failure.. J Heart Lung Transplant.

[20] Chen PM, Leng XG, Fan LL, Ma J, Wang YF (2005). Changes of chymase, angiotensin converting enzyme and angiotensin II type 1 receptor expressions in the hamster heart during the development of heart failure.. Chin Med J (Engl).

[21] Dell'Italia LJ, Meng QC, Balcells E, Straeter-Knowlen IM, Hankes GH (1995). Increased ACE and chymase-like activity in cardiac tissue of dogs with chronic mitral regurgitation.. Am J Physiol.

[22] Dell'Italia LJ, Husain A (2002). Dissecting the role of chymase in angiotensin II formation and heart and blood vessel diseases.. Curr Opin Cardiol.

[23] Huang XR, Chen WY, Truong LD, Lan HY (2003). Chymase is upregulated in diabetic nephropathy: implications for an alternative pathway of angiotensin II-mediated diabetic renal and vascular disease.. J Am Soc Nephrol.

[24] Urata H, Kinoshita A, Misono KS, Bumpus FM, Husain A (1990). Identification of a highly specific chymase as the major angiotensin II-forming enzyme in the human heart.. J Biol Chem.

[25] Urata H, Healy B, Stewart RW, Bumpus FM, Husain A (1990). Angiotensin II-forming pathways in normal and failing human hearts.. Circ Res.

[26] Urata H, Boehm KD, Philip A, Kinoshita A, Gabrovsek J (1993). Cellular localization and regional distribution of an angiotensin II-forming chymase in the heart.. J Clin Invest.

[27] Wei CC, Hase N, Inoue Y, Bradley EW, Yahiro E (2010). Mast cell chymase limits the cardiac efficacy of Ang I-converting enzyme inhibitor therapy in rodents.. J Clin Invest.

[28] Akasu M, Urata H, Kinoshita A, Sasaguri M, Ideishi M (1998). Differences in tissue angiotensin II-forming pathways by species and organs in vitro.. Hypertension.

[29] Balcells E, Meng QC, Johnson WH, Oparil S, Dell'Italia LJ (1997). Angiotensin II formation from ACE and chymase in human and animal hearts: methods and species considerations.. Am J Physiol.

[30] Becari C, Teixeira FR, Oliveira EB, Salgado MC (2011). Angiotensin-converting enzyme inhibition augments the expression of rat elastase-2, an angiotensin II-forming enzyme.. Am J Physiol Heart Circ Physiol.

[31] Urata H, Kinoshita A, Perez DM, Misono KS, Bumpus FM (1991). Cloning of the gene and cDNA for human heart chymase.. J Biol Chem.

[32] Kinoshita A, Urata H, Bumpus FM, Husain A (1991). Multiple determinants for the high substrate specificity of an angiotensin II-forming chymase from the human heart.. J Biol Chem.

[33] Fleming I (2006). Signaling by the angiotensin-converting enzyme.. Circ Res.

[34] Kielty CM, Lees M, Shuttleworth CA, Woolley D (1993). Catabolism of intact type VI collagen microfibrils: susceptibility to degradation by serine proteinases.. Biochem Biophys Res Commun.

[35] Lee M, Calabresi L, Chiesa G, Franceschini G, Kovanen PT (2002). Mast cell chymase degrades apoE and apoA-II in apoA-I-knockout mouse plasma and reduces its ability to promote cellular cholesterol efflux.. Arterioscler Thromb Vasc Biol.

[36] Tchougounova E, Pejler G (2001). Regulation of extravascular coagulation and fibrinolysis by heparin-dependent mast cell chymase.. FASEB J.

[37] Tchougounova E, Pejler G, Abrink M (2003). The chymase, mouse mast cell protease 4, constitutes the major chymotrypsin-like activity in peritoneum and ear tissue. A role for mouse mast cell protease 4 in thrombin regulation and fibronectin turnover.. J Exp Med.

[38] Fukami H, Okunishi H, Miyazaki M (1998). Chymase: its pathophysiological roles and inhibitors.. Curr Pharm Des.

[39] Inoue N, Muramatsu M, Jin D, Takai S, Hayashi T (2009). Effects of chymase inhibitor on angiotensin II-induced abdominal aortic aneurysm development in apolipoprotein E-deficient mice.. Atherosclerosis.

[40] Kanemitsu H, Takai S, Tsuneyoshi H, Yoshikawa E, Nishina T (2008). Chronic chymase inhibition preserves cardiac function after left ventricular repair in rats.. Eur J Cardiothorac Surg.

[41] Komeda K, Jin D, Takai S, Hayashi M, Takeshita A (2008). Significance of chymase-dependent angiotensin II formation in the progression of human liver fibrosis.. Hepatol Res.

[42] Miyazaki M, Takai S (2006). Tissue angiotensin II generating system by angiotensin-converting enzyme and chymase.. J Pharmacol Sci.

[43] Takai S, Shiota N, Sakaguchi M, Muraguchi H, Matsumura E (1997). Characterization of chymase from human vascular tissues.. Clin Chim Acta.

[44] Takai S, Jin D, Sakaguchi M, Miyazaki M (1999). Chymase-dependent angiotensin II formation in human vascular tissue.. Circulation.

[45] Takai S, Jin D, Miyazaki M (2010). New approaches to blockade of the renin-angiotensin-aldosterone system: chymase as an important target to prevent organ damage.. J Pharmacol Sci.

[46] Miyazaki M, Takai S, Jin D, Muramatsu M (2006). Pathological roles of angiotensin II produced by mast cell chymase and the effects of chymase inhibition in animal models.. Pharmacol Ther.

[47] Nakajima K, Powers JC, Ashe BM, Zimmerman M (1979). Mapping the extended substrate binding site of cathepsin G and human leukocyte elastase. Studies with peptide substrates related to the alpha 1-protease inhibitor reactive site.. J Biol Chem.

[48] Skeggs LT, Levine M, Lentz KE, Kahn JR, Dorer FE (1977). New developments in our knowledge of the chemistry of renin.. Fed Proc.

[49] Dorer FE, Kahn JR, Lentz KE, Levine M, Skeggs LT (1975). Formation of angiotensin II from tetradecapeptide renin substrate by angiotensin-converting enzyme.. Biochem Pharmacol.

[50] Dell'Italia LJ, Rocic P, Lucchesi PA (2002). Use of angiotensin-converting enzyme inhibitors in patients with diabetes and coronary artery disease.. Curr Probl Cardiol.

[51] Calkins H, Brugada J, Packer DL, Cappato R, Chen SA (2007). HRS/EHRA/ECAS expert Consensus Statement on catheter and surgical ablation of atrial fibrillation: recommendations for parsonnel, policy, procedures and follow-up. A report of the Heart Rhythm Society (HRS)Task Force on catheter and surgical ablation of atrial fibrillation.. Heart Rhythm.

[52] Gwathmey TM, Shaltout HA, Pendergrass KD, Pirro NT, Figueroa JP (2009). Nuclear angiotensin II type 2 (AT2) receptors are functionally linked to nitric oxide production.. Am J Physiol Renal Physiol.

[53] Shaltout HA, Westwood BM, Averill DB, Ferrario CM, Figueroa JP (2007). Angiotensin metabolism in renal proximal tubules, urine, and serum of sheep: evidence for ACE2-dependent processing of angiotensin II.. Am J Physiol Renal Physiol.

[54] Cohen JA, Lindsey SH, Pirro NT, Brosnihan KB, Gallagher PE (2010). Influence of estrogen depletion and salt loading on renal angiotensinogen expression in the mRen(2).Lewis strain.. Am J Physiol Renal Physiol.

